# Geriatric Interprofessional Education Immersion: A Student Perspective Applying the 4Ms

**DOI:** 10.1093/geroni/igaf122.3945

**Published:** 2025-12-31

**Authors:** Grace Mulligan, Jezebel Ortiz Villaman, Nelson Thomas, Maud Formson, Emilia Sanchez, Emily Balog

**Affiliations:** Rowan University, Stratford, New Jersey, United States; Rutgers University, Camden, New Jersey, United States; Rowan University School of Osteopathic Medicine, Stratford, New Jersey, United States; Thomas Jefferson University, Philadelphia, Pennsylvania, United States; Rutgers University, Camden, New Jersey, United States; Rowan University School of Osteopathic Medicine, Stratford, New Jersey, United States

## Abstract

The U.S. healthcare system is not prepared to address the complex needs of the older adult population nearing 78 million by 2050. This requires a proactive shift toward age-friendly healthcare systems. The Age-Friendly Health Systems initiative developed the 4Ms framework: What Matters, Mentation, Medication, and Mobility which seeks to address the geriatric workforce needs. This poster presents how an innovative geriatric education and training program influenced interdisciplinary students’ understanding and application of person-centered care. Over eight weeks, medical, nursing, social work, and occupational therapy students participated in a 160-hour immersion that combined didactic sessions, community site visits, and application at a Fair Share Housing community in Camden, NJ (i.e., the interprofessional supersite). Students integrated knowledge through hands-on experience, developing interventions responsive to resident needs at the interprofessional supersite. Key themes included the importance of identifying patient values (What Matters), understanding cognitive and social engagement needs (Mentation), addressing polypharmacy and food-as-medicine (Medication), and assessing fall risks, transportation, and accessibility challenges (Mobility). Students also gained insights into how social determinants and systemic disparities influence health equity across communities. Interprofessional collaboration and cultural humility emerged as essential elements for personal and professional growth necessary for delivering effective, compassionate care. This educational model demonstrated that early exposure to age-friendly care fosters critical thinking, enhances empathy, and prepares students to deliver patient-centered, interdisciplinary care for a growing aging population. The program’s outcomes support embedding the 4Ms into clinical training to create a workforce prepared to meet the evolving demands of geriatric medicine.

